# Individual differences in compliance and agreement for sleep logs and wrist actigraphy: A longitudinal study of naturalistic sleep in healthy adults

**DOI:** 10.1371/journal.pone.0191883

**Published:** 2018-01-29

**Authors:** Steven M. Thurman, Nick Wasylyshyn, Heather Roy, Gregory Lieberman, Javier O. Garcia, Alex Asturias, Gold N. Okafor, James C. Elliott, Barry Giesbrecht, Scott T. Grafton, Sara C. Mednick, Jean M. Vettel

**Affiliations:** 1 U.S. Army Research Laboratory, Human Research & Engineering Directorate, Aberdeen Proving Ground, Maryland, United States of America; 2 University of Pennsylvania, Department of Bioengineering, Philadelphia, Pennsylvania, United States of America; 3 University of California, Santa Barbara, Department of Psychological & Brain Sciences, Santa Barbara, California, United States of America; 4 University of California, Irvine, Department of Cognitive Science, Irvine, California, United States of America; Associazione OASI Maria SS, ITALY

## Abstract

There is extensive laboratory research studying the effects of acute sleep deprivation on biological and cognitive functions, yet much less is known about naturalistic patterns of sleep loss and the potential impact on daily or weekly functioning of an individual. Longitudinal studies are needed to advance our understanding of relationships between naturalistic sleep and fluctuations in human health and performance, but it is first necessary to understand the efficacy of current tools for long-term sleep monitoring. The present study used wrist actigraphy and sleep log diaries to obtain daily measurements of sleep from 30 healthy adults for up to 16 consecutive weeks. We used non-parametric Bland-Altman analysis and correlation coefficients to calculate agreement between subjectively and objectively measured variables including sleep onset time, sleep offset time, sleep onset latency, number of awakenings, the amount of wake time after sleep onset, and total sleep time. We also examined compliance data on the submission of daily sleep logs according to the experimental protocol. Overall, we found strong agreement for sleep onset and sleep offset times, but relatively poor agreement for variables related to wakefulness including sleep onset latency, awakenings, and wake after sleep onset. Compliance tended to decrease significantly over time according to a linear function, but there were substantial individual differences in overall compliance rates. There were also individual differences in agreement that could be explained, in part, by differences in compliance. Individuals who were consistently more compliant over time also tended to show the best agreement and lower scores on behavioral avoidance scale (BIS). Our results provide evidence for convergent validity in measuring sleep onset and sleep offset with wrist actigraphy and sleep logs, and we conclude by proposing an analysis method to mitigate the impact of non-compliance and measurement errors when the two methods provide discrepant estimates.

## Introduction

Sleep is a fundamental biological need that impacts cognition and behavior [1–4], with specific effects on the regulation of mood [5], attention [6–9], memory [10], and emotion [11,12]. Transient episodes of sleep deprivation are associated with a variety of functional deficits [13,14], and chronic sleep loss may have an even more adverse and sustained impact on health, mood, and behavior over time [15–18]. While many laboratory studies have examined the impact of acute sleep deprivation (> 24 hours) on vigilance and cognitive performance, much less is known about real-world sleep variability and how it might affect fluctuations in behavior and performance over time [19,20]. To better understand how naturalistic sleep variability impacts behavior, it is first necessary to evaluate current tools for collecting longitudinal measurements of sleep over extended periods of time, and with minimal intrusion in their normal sleeping environment.

Although polysomnography (PSG) is the generally accepted gold standard for objective measurement of sleep states based on oscillatory signals in the brain, muscle activity, and cardiopulmonary patterns [21–23], it is methodologically too intrusive and research-intensive for long-term studies of naturalistic sleep. Instead, longitudinal sleep studies must rely on indirect inferential methods such as sleep log diaries [24], questionnaires that are based on an individual’s memory about the previous night’s sleep [25], and wrist actigraphy, which uses accelerometry to measure body movement and infer wake and sleep states from levels of activity/inactivity with specialized scoring algorithms [26,27]. Many studies have compared wrist actigraphy to PSG and concluded that actigraphy can be useful in distinguishing sleep versus wake states [28–30]. These studies generally find suitable agreement for variables such as sleep onset and total sleep time [22,31–35] but decidedly less agreement in identifying transitions between sleep and wakefulness during the sleep period [35,36]. Nonetheless, wrist actigraphy is widely regarded as a valid and reliable tool for measuring the macrostructure of sleep, including broad transitions between sleep and wakefulness (e.g. sleep onset and sleep offset) in healthy adult populations [29,36].

Most of the experimental literature to date has tended to examine real-world sleep variability over short periods of time, up to approximately two weeks [37], which limits our understanding of the efficacy and potential compliance issues associated with longer timescale sleep measurements. The present study measured naturalistic sleep variables derived from sleep logs and wrist actigraphy from 30 individuals for up to 16 consecutive weeks. The long-term study design afforded three complementary analyses. We first assessed the level of agreement between actigraphy and sleep logs for estimating variables related to sleep and wake states, expecting some level of consistency between the two methods [29,36], but also hypothesizing potential individual differences [38] across the different types of sleep metrics [39]. Second, we examined task compliance in terms of submitting sleep logs daily according to experimental instructions, expecting that compliance would tend to worsen over time [40,41], but also hypothesizing individual differences in the ability of participants to sustain motivation and achieve strict compliance for four consecutive months. Last we examined the relationship between compliance and agreement, evaluating whether individuals showing higher compliance also tended to produce higher fidelity subjective estimates of their sleep with reference to objective actigraph measurements. We conclude by proposing a model that combines actigraphy and sleep log measurements to produce a singular robust estimate of sleep variables, even when the two methods provide discrepant estimates.

## Material and method

### Participants

Thirty healthy participants (mean age = 23.0 years, age range 18–35 years, 13 males, 17 females) were recruited by word of mouth and local advertisements. The University of California, Santa Barbara (UCSB) Human Subjects Committee (#16–0154) and Army Research Laboratory Human Research Protections Office (#14–098) approved all procedures, and all participants provided informed written consent. Research was conducted in accordance with the declarations of Helsinki. The data presented in this manuscript represent a subset of data collected as part of a large-scale, longitudinal experimental protocol called Cognitive Resilience and Sleep History (CRASH) that collected bi-weekly structural and functional brain data, peripheral physiology, eye-tracking, blood and saliva samples. Neuroimaging and physiological data were collected bi-weekly to investigate how sleep history modulates the relationship between physiology and performance. The present work has specific focus on the foundational question of how to characterize sleep history from distinct data types (sleep logs and actigraphy) collected across 16 consecutive weeks in a natural environment.

### Protocol

Prior to participation in the main experiment, participants completed personality trait questionnaires including the big five inventory [42] and the behavioral avoidance and behavioral approach scale [43]. The big five inventory (BFI) assessed personality along five standard trait dimensions including extroversion (BFI-E), agreeableness (BFI-A), conscientiousness (BFI-C), neuroticism (BFI-N), and openness (BFI-O). The behavioral avoidance/approach scale (BIS/BAS) assessed motivational traits including behavioral inhibition (BIS) and 3 sub-scales of behavioral approach including drive (BAS-D), fun seeking (BAS-F), and reward responsiveness (BAS-R). Responses to questionnaire items were scored according to standard procedures [42,43].

Participants were instructed to complete daily sleep log questionnaires upon awakening using the wake-time component of the Pittsburgh Sleep Diary [25] and an online survey display (Qualtrics, version: September, 2016, Provo, Utah) that provided a digital time stamp to confirm when the survey was started and completed. Participants reported five metrics about their sleep history: when they went to bed to initiate sleep, how long it took to fall asleep (sleep onset latency), when they woke up (sleep offset), how many times they woke up during the night (number of nightly awakenings), and how many minutes were spent awake during those awakenings (wake after sleep onset).

Participants were instructed to wear a wrist actigraph device continuously throughout the study, with the exception of taking off the device during biweekly laboratory visits (approximately 3 hours in duration). During the biweekly visits, researchers uploaded the data from the actigraph watch and made sure the watch was charged and functioning properly. The participants were scheduled to return to the lab for eight bi-weekly sessions, so the duration of recorded sleep measurements lasted approximately 16 weeks (112 days) for each participant. However, there was some variability in the total number of days measured due to scheduling issues, scanner malfunctions, travel, and holidays. Therefore, we only consider data from the first day of the study up to at most 112 consecutive days (even if data collection continued past 16 weeks) to facilitate group analysis on a commensurate timeline.

### Analysis

#### Sleep log data processing

Subjective responses to the Pittsburgh Sleep Diary items allowed estimation of six variables that represent metrics of sleep quality and sleep quantity: sleep onset time (SON), sleep offset time (SOFF), sleep onset latency (SOL), wake after sleep onset (WASO), number of nightly awakenings (NNA), and total sleep time (TST). Sleep onset was defined as the self-reported time the individual went to bed to initiate sleep, plus the self-reported time it took to fall asleep (i.e., SOL). Wake after sleep onset represents the self-reported total number of minutes spent awake during all nightly awakenings. The sleep period is defined as the time interval from sleep onset to sleep offset, and total sleep time represents total hours spent asleep during the sleep period after subtracting WASO (TST = SOFF–SON–WASO).

#### Actigraphy data processing

Actigraph data were acquired continuously with a Readiband Actigraph SBV2 (Fatigue Science, Vancouver, BC). This device measures movement using a 3D accelerometer sampled at 16 Hz and stores the data internally. The raw output of the device was processed by Fatigue Science software to estimate two discrete variables at every minute of the day: 1) whether the individual was “in bed” or “out of bed” and 2) whether the individual was “asleep” or “awake.” The Readiband device has been validated with respect to polysomnography in a white paper on the company’s website [44], and has been evaluated for internal consistency [45], as well as consistency with self-reported sleep data in rugby players [46] and in use for feedback to affect sleep hygiene of soldiers [47]. We define sleep onset as the first recorded instance of sleep occurring at or after 9:00pm, unless the participant was asleep at 9:00pm, in which case we use the latest transition from wake to sleep prior to 9:00pm, in accordance with the advice of Berger et al. [48]. Sleep offset was defined as the last instance of transitioning from sleep to wake before 11:00am on the following day; however, if the person was still labeled as asleep at 11:00am, then the next transition from sleep to wake was considered as the sleep offset. The work by Berger et al. recommended 9:00am as the cutoff for sleep offset time [48], but 40.0% of participants in our sample reported waking up after 9:00am on their sleep logs, whereas only 8.9% were reported later than 11:00am, so we adapted this recommendation to our participants’ sleeping habits. Sleep onset latency was defined as the difference between the first minute labeled by the model as “in bed” and “awake” and the first minute labeled as “in bed” and “asleep.” A nightly awakening was defined as a transition from asleep to awake during the sleep period. Wake after sleep onset was computed as the total number of wakeful minutes accumulated across all nightly awakenings. Analogous to sleep logs, total sleep time was computed as the length of the sleep period minus the amount of time awake during the sleep period (TST = SOFF–SON–WASO). All sleep/wake variables were measured to the nearest minute and are reported here in hours for consistency in units across measures.

#### Agreement between actigraphy and sleep logs

Sleep/wake variables derived from actigraphy and sleep logs are presumed to originate from the same objective sleep/wake experience of the individual. However, each methodology depends on fundamentally different source data to infer these values. Sleep logs rely upon the memory of the individual about their sleep/wake experience of the previous night, whereas actigraphy infers wake and sleep epochs from changes in the amount of body activity measured continuously over time from wrist movement. Since each method can capture many of the same types of sleep/wake variables, it is reasonable to expect some degree of agreement between their measurements. On the other hand, since the methods rely on such distinct source data (memory versus wrist movement), there may also be substantial discrepancies in their measurements. A chief aim of our analysis was therefore to characterize which sleep/wake variables showed the most and least agreement and to describe any systematic differences, or biases, between the two methods.

For the group-based analysis, we evaluated agreement across all participants (*n* = 30) and time points (up to 112 days) concatenated to a single vector. There were some days in which participants failed to comply by not completing the sleep log questionnaire, and other instances in which the wrist actigraphy data were missing (e.g., the device ran out of battery or was taken off the wrist), resulting in intermittent episodes of data loss. Sleep log data in which sleep logs were submitted more than 24 hours after awakening were also discarded from this analysis to eliminate the possibility of completing multiple days retrospectively (i.e. hoarding) [49], and to reduce susceptibility to bias and distortions commonly found in retrospective self-reports [50]. Our analysis included only days in which there were valid data from both sleep logs and actigraphy, which included 2,417 days out of 3,307 possible days across participants (73.0% of total data). Furthermore, there were individual differences in sleep log compliance and, as a result, the percentage of days in which sleep logs were submitted ranged from 28.6% to 100% across subjects.

To characterize the level of agreement between the methods, we used two complementary approaches including: 1) computing Pearson correlation coefficients to characterize the relative strength of the relationship between the two measurements, and 2) performing a non-parametric analysis of the distribution of differences between the measurements as recommended by Bland and Altman for situations in which the difference distribution is non-normal [51].

**Fig 1:**
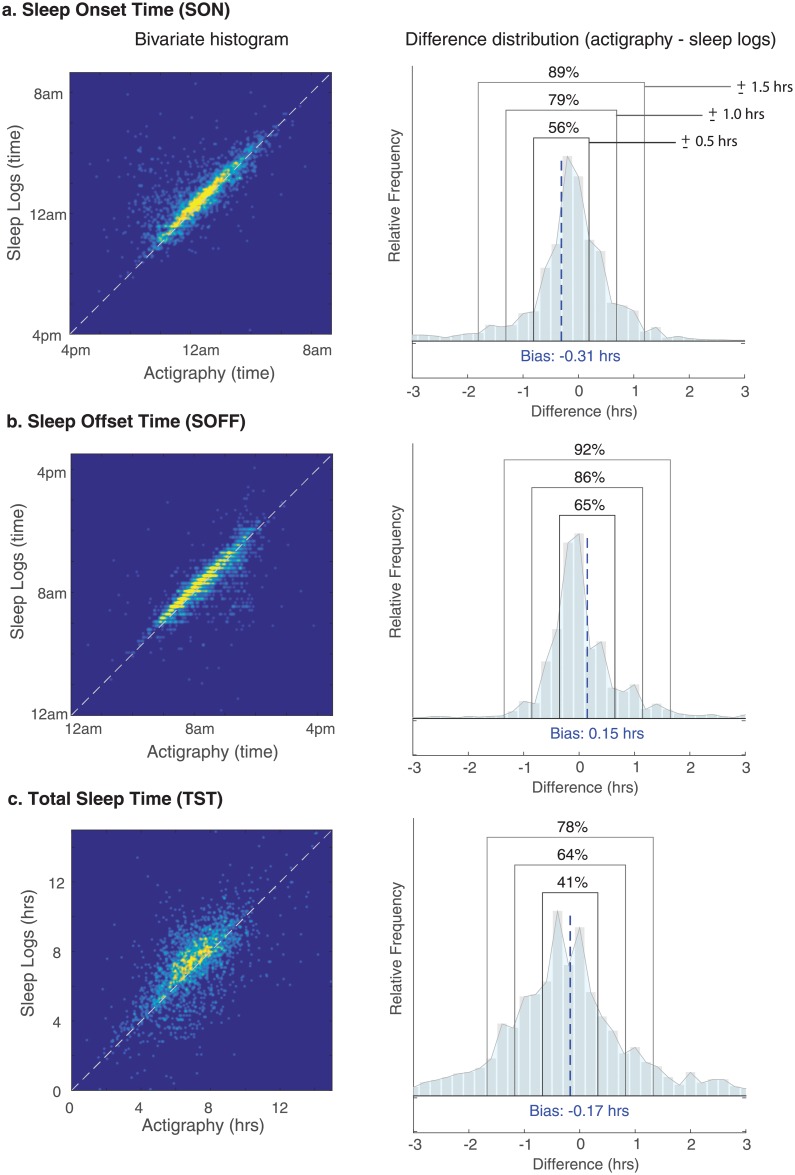
The relationship between daily sleep log and wrist actigraphy measurements. Bivariate histograms for (a) sleep onset (SON), (b) sleep offset (SOFF), and (c) total sleep time (TST). The white dotted line represents the equality line (perfect agreement); therefore, deviations from the line indicate a lack of agreement. The empirical distributions (right panels) show the distribution of differences (actigraphy minus sleep log values), with the black dotted line representing the bias, and boxes demarcating the three reference intervals included in our non-parametric Bland-Altman analysis (±0.5, ±1.0, ±1.5 hours).

The Bland-Altman analysis provides a measure of absolute agreement by quantifying the mean bias and the percentage of data contained within specific reference intervals relative to the bias (e.g., what percentage of empirical differences is contained within ±1 hour). The bias is defined as the mean difference between two measurements, where a bias equal to zero would indicate no difference, and values greater than or less than zero would indicate a directional bias, for example, whether actigraphy tended to produce values that were consistently less than or greater than sleep logs. Since the difference distributions for measurements in our study had sharper peaks and longer tails than expected by chance with reference to the Gaussian distribution, as confirmed by Kilmorgoov-Smirnoff tests for goodness-of-fit of a standard normal distribution (all *p*’s < 0.001), we employed a non-parametric analysis that calculated the percentage of empirical differences (actigraphy–sleep logs) contained within specified reference intervals, including ±0.5, ±1.0 and ±1.5 hours (Fig 1). High agreement is indicated by a larger proportion of data being contained within smaller difference intervals.

Due to the longitudinal design and extensive daily measurements for each participant (*M* = 81 valid days of measurement, *SD* = 22.4), we also had statistical power to assess agreement for individual participants across time. Following the non-parametric Bland-Altman analysis of the group data, we examined the percentage of data from the difference distribution contained within the target interval of ±1 hour across all days for each participant. The percentage of data contained within this interval provides an indicator of the amount of data for which the two measures agree suitably, where we define one hour to be a reasonable and ecologically valid cutoff point specifically for measuring sleep onset and sleep offset times.

#### Sleep log compliance

Participants in this study were asked to complete sleep log questionnaires as soon as possible after awakening, but the time stamps for the online submission of the self-report data were expected to vary both between individuals and within an individual over time. To quantify this variability, we examined compliance in two ways: 1) did the participant submit a sleep log as instructed on a given day, and 2) how soon after awakening was the sleep log submitted?

We defined compliance rate at the group level as the proportion of participants that successfully completed a sleep log on a particular day of the study (e.g., how many participants submitted a sleep log on their first day, on their second day, etc., up to at most 112 days in the study). We defined compliance rate at the individual level as the proportion of days in which the participant successfully submitted sleep logs out of the total number of days they were enrolled in the study (e.g. up to 112 days).

We also examined compliance in terms of the time delay between when the participant self-reported waking up and the time stamp for submitting the sleep log online. Analogous to analysis for compliance rate, time delay at the group level represents the average delay across participants for a given day of the study relative to each person’s start date, and time delay at the individual level represents the average delay across all days for which that participant was enrolled in the study.

Between-subjects relationships between compliance and other factors were assessed with Pearson’s product-moment correlation coefficients. We examined the correlation between compliance variables and the agreement level between sleep logs and actigraphy to examine whether individual differences in agreement were related to differences in overall compliance. To help explain individual differences in compliance, we further examined relationships between compliance variables and personality trait scores derived from the BFI and BIS/BAS questionnaires.

## Results

### Descriptive statistics: Group means

We first compared the measurements of six common metrics that quantify sleep characteristics and that capture the amount of time in bed (sleep onset/SON, sleep offset/SOFF, and total sleep time/TST) and the amount of time awake during the sleep period (number of awakenings/NNA, wake after sleep onset/WASO), as well as the time awake while trying to fall asleep while lying in bed (sleep onset latency/SOL). Group means for sleep/wake variables, measured independently from actigraphy and sleep logs, are reported in Table 1.

**Table 1:** Group mean results for six variables measured by wrist actigraphy and sleep logs.

	Actigraphy		Sleep logs		Pearson	Bland-Altman analysis			
	*m (hrs)*	*sd*	*m (hrs)*	*sd*	*r*	*bias (hrs)*	*0*.*5h*	*1*.*0h*	*1*.*5h*
SON	12:36am	1.96	12:57am	1.77	0.73*	-0.31*	56%	79%	89%
SOFF	8:39am	1.78	8:31am	1.76	0.77*	0.15*	65%	86%	92%
TST	7.09	1.77	7.25	2.02	0.62*	-0.17*	41%	64%	78%
SOL	0.51	0.69	0.28	0.4	0.1*	0.19*	80%	93%	97%
WASO	0.95	1.16	0.31	0.63	0.01	0.63*	45%	80%	86%
	*m (na)*	*sd*	*m (na)*	*sd*	*r*	*bias (na)*	*2na*	*4na*	*6na*
NNA	3.52	2.86	1.45	1.55	0.03	2.12*	46%	81%	93%

We used non-parametric Bland-Altman analysis to characterize the percentage of data contained within reference intervals for variables measured in hours (hrs) ±0.5, ±1.0, ±1.5h, and for nightly awakenings where the units are number of awakenings (na) with intervals of ±2, ±4, ±6na. Abbreviations: sleep onset time (SON), sleep offset time (SOFF), total sleep time (TST), sleep onset latency (SOL), wake after sleep onset (WASO), and number of nightly awakenings (NNA), product-moment correlation coefficient (r).

*p < 0.001.

We found that mean values for SON, SOFF and TST were similar whether derived from actigraphy or sleep logs; but due to the large number of observations, even these relatively small differences were found to be statistically significant as evaluated by t-tests comparing the bias, or mean of the difference distribution (actigraphy–sleep logs), to zero. Actigraphy measurements for SON were 18.5 minutes earlier than measurements from sleep logs (bias = -0.31 hrs, *t*(2415) = -11.1, *p* < 0.001), measurements of SOFF were 8.8 minutes later than sleep logs (bias = 0.15 hrs, *t*(2415) = 6.2, *p* < 0.001), and measurements of TST were 10.3 minutes shorter than sleep logs (bias = -0.17 hrs, *t*(2415) = -5.4, *p* < 0.001), on average.

The two methods diverged more substantially in measuring SOL, WASO, and the NNA. Actigraphy measurements for WASO were on average 37.5 minutes longer than sleep logs (bias = 0.62 hrs, *t*(2415) = 23.1, *p* < 0.001). A likely contributor to this increase in WASO was due to the fact that actigraphy measured significantly more nightly awakenings than sleep logs (bias = 2.12 awakenings, *t*(2415) = 32.5, *p* < 0.001). Actigraphy also produced measurements of SOL that were 11.6 minutes longer on average than sleep logs (bias = 0.19 hrs, *t*(2415) = 23.1, *p* < 0.001). In total, there was a general tendency for actigraphy to produce larger measurements associated with wakefulness (SOL, WASO, NNA) than measurements obtained by self-report, which is consistent with existing literature [28,39].

### General agreement between actigraphy and sleep logs

To assess the strength of relationship between sleep/wake variables derived from actigraphy and sleep logs, we also measured Pearson’s product-moment correlation coefficients. Bivariate histograms (Fig 1 left) illustrate the strength of these relationships across all daily measurements in the study for three variables that showed the greatest agreement (SON, SOFF and TST). There were very strong correlations between actigraphy and sleep logs for SON (Fig 1a; *r* = 0.73, *p* < 0.0001) and SOFF (Fig 1b; *r* = 0.77, *p* < 0.0001), and to a slightly lesser degree TST (Fig 1c; *r* = 0.62, *p* < 0.0001). The correlation for SOL was substantially weaker but still statistically significant (*r* = 0.10, *p* < 0.0001). However, the correlation between actigraphy and sleep logs was not significant for WASO (*r* = 0.01, *p* = 0.7) or NNA (*r* = 0.03, *p* = 0.5). These results demonstrate convergence between actigraphy and sleep logs in measuring when individuals fell asleep and woke up, but less consistency in measuring the frequency and duration of awakenings.

Agreement was quantified by calculating the percentage of the empirical data contained within three specific reference intervals (Table 1), while taking into account the mean difference by centering the interval on the bias between the measurements (Fig 1 right). For sleep/wake variables that were measured in units of hours (SON, SOFF, TST, SOL, WASO), we define three intervals to span a reasonable range of differences (±0.5, ±1.0, ±1.5 hours). The number of nightly awakenings (NNA) was not measured in hours, so we specify a reasonable set of reference intervals for this variable separately (±2, ±4, ±6 awakenings).

The best agreement was found for SOFF, in which 65% of differences were within ±0.5 hours and 92% were within ±1.5 hours (Fig 1a), and for SON, in which 56% of differences were within ±0.5 hours and 89% were within ±1.5 hours (Fig 1b). TST also showed reasonable agreement considering the magnitude of this variable (*M* = 7.17 hrs), with 41% of differences falling within ±0.5 hours and 78% of differences falling within ±1.5 hours (Fig 1c). As can be seen in Fig 1, these difference distributions are characterized by a rather sharp peak in the center, indicating that a majority of differences are contained in a narrow interval of about ±1 hour, but also by relatively long tails, indicating that a small percentage of days showed extremely divergent measurements greater than ±2 hours. Such discrepancies between actigraphy and sleep logs on this minor subset of data may represent cases in which, for one reason or another, one of the methods produced a high error measurement, for example, due to memory failure, lack of motivation or effort, or possibly due to a scoring error in processing raw actigraph data.

Overall, there was less agreement for variables that measured night wakefulness in relation to the overall magnitude of these variables. We found for sleep onset latency that 80% of the differences were within ±0.5 hours, and 96% of differences were within ±1.5 hours. However, the group mean SOL values were only 0.51 and 0.28 hours for actigraphy and sleep logs, respectively, so the fact that only 80% of differences were within ±0.5 hours could hardly be characterized as strong agreement. For wake after sleep onset, we found that only 45% of differences were within ±0.5 hours and 86% of differences were within ±1.5 hours, despite the fact that mean WASO values were only 0.95 and 0.31 hours for actigraphy and sleep logs, respectively. This reveals a substantial discrepancy between actigraphy and sleep logs in measuring WASO, which is consistent with the lack of a linear correlation found between the methods for WASO and NNA. For the number of nightly awakenings, 46% of differences were within ±2 awakenings and 92% of differences were within ±6 awakenings; yet, mean NNA values were only 3.5 and 1.4 awakenings for actigraphy and sleep logs, respectively.

The discrepancy between actigraphy and sleep logs for variables representing how often and for how long individuals were awake at night while in bed (SOL, WASO, NNA) may be attributed to differences in sensitivity of the two measurements. For example, people may be more likely to recollect only substantial or salient awakenings, whereas actigraphy may have better sensitivity in detecting brief episodes corresponding to detection of subtle wrist movements. While self-report could plausibly lead to errors by under-reporting brief wake episodes, the heightened sensitivity of actigraph could also lead to errors in overestimating these events.

When considered together, these results provide converging evidence from actigraphy and sleep logs in measuring SON and SOFF times. Since these were the principle variables demonstrating both strong correlations and good agreement within our dataset, subsequent analyses in this paper will focus on 1) characterizing individual differences in agreement for SON and SOFF variables, 2) examining the relationship between agreement and task compliance across individuals, and 3) developing a framework for combining these independent measurements to provide a *best estimate* of the theoretical (but unobserved) ground truth values associated with SON and SOFF times.

### Individual differences in agreement

**Fig 2:**
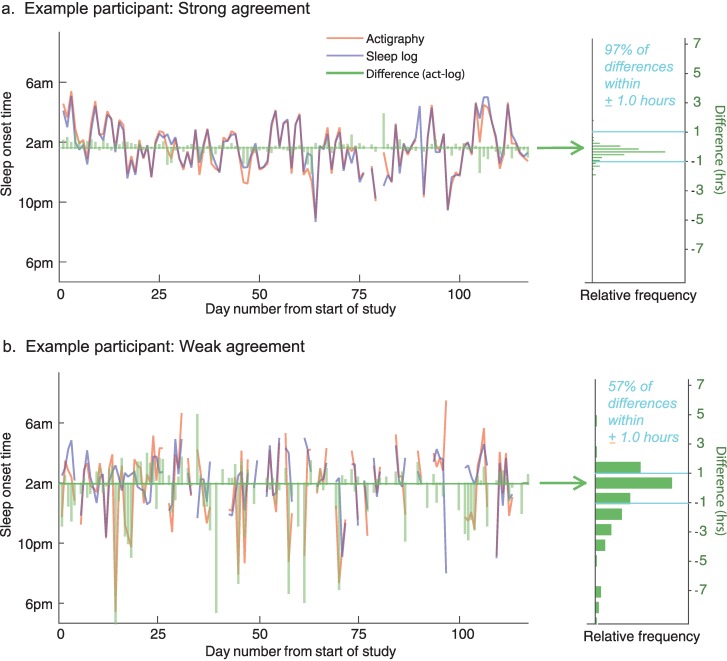
Example data comparing daily sleep onset times from actigraphy and sleep logs. The time series’ in (a) show a participant with strong agreement, and (b) show a participant with relatively weak agreement. Green bars represent the difference between the measures (actigraphy–sleep log). The right panels illustrate difference distributions across all measurements for that participant.

We examined individual differences in agreement between actigraphy and sleep logs for SON and SOFF times. If actigraphy and self-reported sleep logs tended to produce similar measurements for an individual over time, this would be reflected in a higher percentage of differences being contained within the target reference interval of ±1 hour. An example of an individual participant with strong agreement is shown in Fig 2a, in which 97% of absolute differences were less than ±1 hour. However, several individuals showed far worse agreement than this. For example, the participant shown in Fig 2b had only 57% of differences within one hour and had several measurements in which actigraphy underestimated sleep onset by more than 3 hours in comparison to self-report. Table 2 shows agreement levels for all individuals along with compliance rates and demographic characteristics of our sample.

**Table 2:** Individual differences in bias, agreement, compliance and demographic variables.

Subject	bias (SON)	Agree. (SON)	bias (SOFF)	Agree. (SOFF)	Compl.	Delay	Age	Gender
1	-0.15	0.96	-0.06	0.99	1.00	0.94	20	f
2	-0.04	0.97	-0.05	0.90	0.64	3.20	24	f
3	-0.38	0.94	-0.01	0.95	1.00	0.38	22	f
4	-0.09	0.78	0.47	0.82	0.76	3.99	35	f
5	-0.40	0.65	0.32	0.85	0.50	3.79	25	m
6	-0.38	0.77	-0.10	0.83	0.46	4.33	20	m
7	-0.26	0.75	-0.27	0.78	0.77	2.75	22	f
8	0.01	0.95	-0.26	0.89	0.86	2.78	23	f
9	-0.35	0.87	0.12	0.86	0.95	2.36	21	f
10	-0.29	0.83	0.08	0.79	0.92	2.39	21	f
11	-0.26	0.81	-0.10	0.92	0.86	3.13	21	m
12	-0.76	0.66	0.59	0.69	0.29	4.98	26	f
13	0.03	0.57	0.88	0.56	0.56	5.97	35	m
14	-0.01	0.86	0.28	0.78	0.79	2.16	21	m
15	0.01	0.90	-0.12	0.90	0.70	3.30	24	m
16	-0.31	0.89	0.00	0.93	0.50	2.94	20	f
17	-0.81	0.49	0.81	0.40	0.71	2.12	22	f
18	-0.03	0.85	-0.13	0.96	0.93	0.70	21	f
19	-0.58	0.83	0.26	0.92	0.88	1.77	20	m
20	-0.81	0.73	0.12	0.89	0.91	2.56	21	f
21	-0.01	0.94	-0.04	0.89	0.98	0.75	21	f
22	-0.70	0.53	1.00	0.54	0.79	1.95	26	m
23	-0.55	0.80	-0.27	0.85	0.66	1.94	22	m
24	-0.03	0.92	-0.22	0.91	0.99	2.92	22	m
25	-0.51	0.82	0.37	0.88	0.66	1.72	26	f
26	-0.36	0.77	0.89	0.60	0.57	4.14	21	m
27	-0.05	0.89	0.21	0.93	0.80	0.48	21	f
28	-0.15	0.77	0.37	0.83	0.63	2.70	22	m
29	-0.66	0.67	0.04	0.86	0.88	0.15	25	m
30	-0.63	0.74	0.41	0.90	0.97	0.38	21	f

Abbreviations: sleep onset time (SON), sleep offset time (SOFF), agreement (Agree., defined as the proportion of differences contained within the reference interval of ±1hr), compliance (Compl., defined as the proportion of days in which a sleep log was successfully submitted), and mean time delay (Delay, defined as the mean difference between self-reported sleep offset and the digital time stamp for submitting sleep logs online).

Interestingly, this variability was quite consistent across the two variables. We found a statistically significant correlation for level of agreement between SON and SOFF across individuals (*r*(29) = 0.80, *p* < 0.0001), demonstrating that individuals with low agreement on one variable also tended to show low agreement on the other variable. Thus, individual differences in agreement appear to be trait-like due to their consistency across measurements.

### Relationship between agreement and compliance

**Fig 3:**
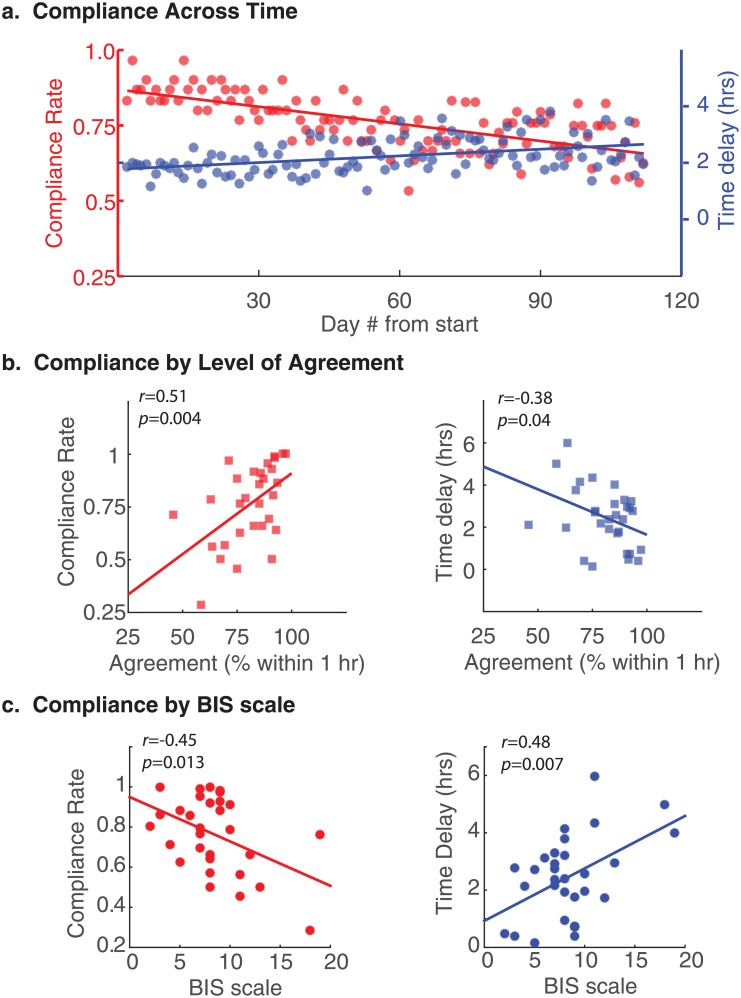
Compliance across time and in relation to agreement and personal traits. (a) Group compliance is shown across the 16-week data collection. The proportion of participants who completed the daily questionnaire is shown in red, and the amount of time delay between self-reported sleep offset and sleep log submission time is shown in blue. Solid lines represent best-fit linear models to the data. (b) Scatter plots illustrate the relationship between an individual’s mean compliance rate (left) and time delay (right) and their level of agreement (SON and SOFF combined). Agreement was defined as the percentage of data with an absolute difference less than one hour. (c) Scatter plots illustrate compliance rate and time delay in relation to behavioral avoidance measures derived from BIS/BAS scale.

We next examined compliance across the 16-week study interval (Table 2). We computed the group-level compliance rate as the proportion of participants that submitted a sleep log for each consecutive day of the study starting from day one up to day 112 (Fig 3a, red). We also computed the average submission delay, representing the time difference from self-reported awakening to the time the sleep log was stamped as submitted to the online system (Fig 3a, blue). We fit linear models to compliance data over time to evaluate the slope and intercept of the model.

We found significant linear trends indicating a general reduction in group compliance rate over time (intercept = 0.87 ± 0.01, *p* < 0.0001; slope = -0.002 ± 0.0002, *p* < 0.0001), and an increase in mean submission delay over time (intercept = 1.77 hrs ± 0.11, *p* < 0.0001; slope = 0.01 ± 0.002, *p* < 0.0001), revealing that compliance tended to worsen significantly over time. At the beginning of the study, approximately 87% of participants completed their daily sleep logs, but by the end of 16 weeks, only 66% of participants remained compliant. Likewise, on average participants completed sleep logs 1.77 hrs after awakening at the beginning of the study, but 2.66 hrs after awakening by the end of week 16. By contrast, the compliance rate for actigraphy was 95.1% across the entire data set, and there was no significant change over time (intercept = 0.95; slope = 0 ± 0.0002, *p* = 0.77).

Next, we examined whether individual differences in compliance could explain some of the variance associated with differences in agreement between actigraphy and sleep logs. The mean within-subjects compliance rates ranged from 0.29 to 1.00 across all days in which each participant was enrolled in the study. Results in Fig 3b reveal a statistically significant correlation (*r* = 0.51, *p* = 0.004) between compliance rate and the level of agreement for SON and SOFF, which were combined to a single metric due to the strong correlation between these variables and their trait-like consistency, as noted above. This linear relationship indicates that individuals with better sustained compliance to the reporting requirements of the study were also more likely to show higher quantitative agreement. There was also a significant relationship between agreement and time delay measurements (Fig 3b), showing that individuals who submitted sleep logs sooner on average after awakening also tended to show significantly stronger levels of agreement (*r* = -0.38, *p* = 0.04). These results suggest that the disagreement between actigraphy and sleep logs for some individuals may be explained, in part, by the fact that these same individuals tended to be less compliant overall with sleep log submission requirements of the study.

### Relationship between compliance and personality traits

Finally, we examined linear relationships between compliance variables and personality trait scores on subscales of the big five inventory (BFI-Extroversion, BFI-Agreeableness, BFI-Conscientiousness, BFI-Neuroticism, BFI-Openness), as well as the BIS/BAS scale (BIS, BAS-Drive, BAS-Fun seeking, BAS-Reward responsiveness). Correlation coefficients across all paired variables are reported in Table 3. As shown in Fig 3c, we found a statistically significant relationship between compliance and behavioral avoidance, or inhibition (BIS), for compliance rate (*r*(29) = -0.45, *p* = 0.013) and mean time delay (*r*(29) = 0.48, *p* = 0.007). Individuals that showed better overall compliance tended to have lower BIS scores, indicating an influence of behavioral avoidance and motivational systems on the propensity for individual compliance to our long-term protocol involving daily questionnaires. None of the other personality trait variables showed a significant relationship to compliance variables (all *p’s* > 0.18).

**Table 3:** Pearson’s correlation coefficients for compliance variables and personality traits.

	Compliance Rate		Mean Time Delay	
Scale	*r(29)*	*pval*	*r(29)*	*pval*
BIS	**-0.45**	0.01*	**0.48**	0.007*
BAS-D	0.16	0.40	-0.06	0.76
BAS-F	-0.07	0.71	0.00	1.00
BAS-R	0.01	0.97	-0.02	0.92
BFI-E	0.24	0.19	-0.08	0.69
BFI-A	-0.07	0.71	-0.20	0.28
BFI-C	0.19	0.30	-0.17	0.36
BFI-N	0.25	0.18	-0.24	0.21
BFI-O	0.14	0.48	-0.16	0.39

Abbreviations: behavioral avoidance scale (BIS), behavioral approach scale–drive (BAS-D), behavioral approach scale–fun seeking (BAS-F), behavioral approach scale–reward responsiveness (BAS-R), big five–extroversion (BFI-E), big five–agreeableness (BFI-A), big five–conscientiousness (BFI-C), big five–neuroticism (BFI-N), big five–openness (BFI-O).

### Proposed method to combine sleep measurements

Although there is generally strong agreement between SON and SOFF measurements at the group level, our results have identified a subset of cases in which there is extreme disagreement greater than two hours (illustrated by the long tails of the difference distributions), as well as consistent individual differences in the discrepancies between the measurement modalities. Here, we introduce a method to help mitigate these discrepancies. This mitigation strategy is a critical component of studying naturalistic sleep loss since it allows a singular and perhaps more robust estimate of SON and SOFF variables.

**Fig 4:**
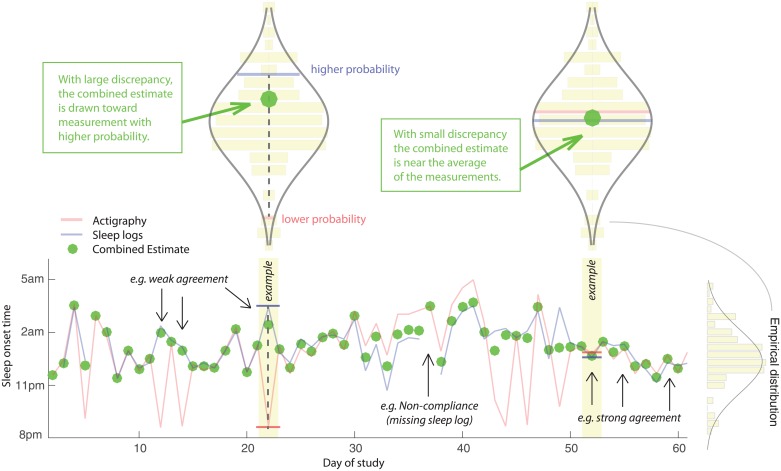
Time series for an example participant showing actigraphy, sleep logs, and model output. Green dots represent the combined estimate for sleep onset time, which was computed as a weighted sum of the actigraphy and sleep log measurements. The text with arrows highlight days exemplifying good agreement, poor agreement, and non-compliance due to a missing sleep log. The upper violin-style plots represent the empirical distribution of all sleep onset measurements from actigraphy and sleep logs. The model was designed for robustness in cases where actigraphy and sleep logs have discrepant measurements (upper left) by producing a composite estimate closer to the more likely value considering the individual’s sleep history. When measurements have similar probability (upper right), the model produces a value that is near the average of the two measurements.

In short, our method combines independent measurements from actigraphy and sleep logs such that convergence on the same value is taken as relatively strong evidence for the true underlying SON or SOFF time. However, when the two estimates diverge, they are weighted according to their likelihood based on the empirical distribution of all measurements for that variable across the sleep history of the individual. Thus, the algorithm for combining values was designed to have the effect of “pulling” divergent measurements toward the more likely of the two measurements, agnostic about whether the value was derived from actigraphy or sleep logs (Fig 4).

For each variable separately (SON and SOFF), we approximated the empirical distribution of measurements across all days derived from both sources (actigraphy and sleep logs) as a normal distribution,

$$X_{Ai},X_{Qi}\;\sim \;N(\bar{x},s^{2})$$

where $\bar{x}$ is defined as the sample mean and *s*^2^ is the sample variance (Fig 4, see violin-style plots). On a given day of the study, represented by index *i*, there will either be two estimates derived from both actigraphy and sleep logs, or there will be a single measurement when data is missing from one of the sources (e.g. often due to a missing self-report). On some occasions, there may be no estimates from actigraphy or sleep logs, and these cases are excluded. When there is data available from only one source, we accept that value as the daily estimate for that sleep variable. However, when data are available at day *i* from both actigraphy, *x*_*Ai*_ and sleep logs, *x*_*Qi*_ we calculate the combined estimate ${\hat{x}}_{comb}$ as the weighted sum of these estimates:

$${\hat{x}}_{comb}\;=\;w_{Ai}x_{Ai}+w_{Qi}x_{Qi}$$

where weights *w*_*Ai*_ and *w*_*Qi*_ are determined by the ratio of the relative probabilities associated with the measurements, based on the empirical distribution of sleep history:

$$w_{Ai}\;=\;\frac{P(x_{Ai})}{P(x_{Ai})+P(x_{Qi})}$$

$$w_{Qi}\;=\;\frac{P(x_{Qi})}{P(x_{Qi})+P(x_{Ai})}$$

The output of the algorithm is illustrated with sample data in Fig 4 (green markers). As shown in the bottom right of the graph; when the two sources are in good agreement then the combined estimate is roughly the average of the two values. However, when the two sources disagree strongly, which is often caused by one source or the other providing a highly improbable (e.g. outlier) estimate, the algorithm produces a combined estimate that is much closer to the value that is most consistent with the individual’s sleep history. This algorithm is designed to deliver robustness for exactly those cases in which one source produced an outlier or uncharacteristic SON or SOFF time; otherwise, it produces roughly an average estimate without inherently favoring actigraphy or sleep logs since the empirical distribution is cast across all measurements from both sources.

**Fig 5:**
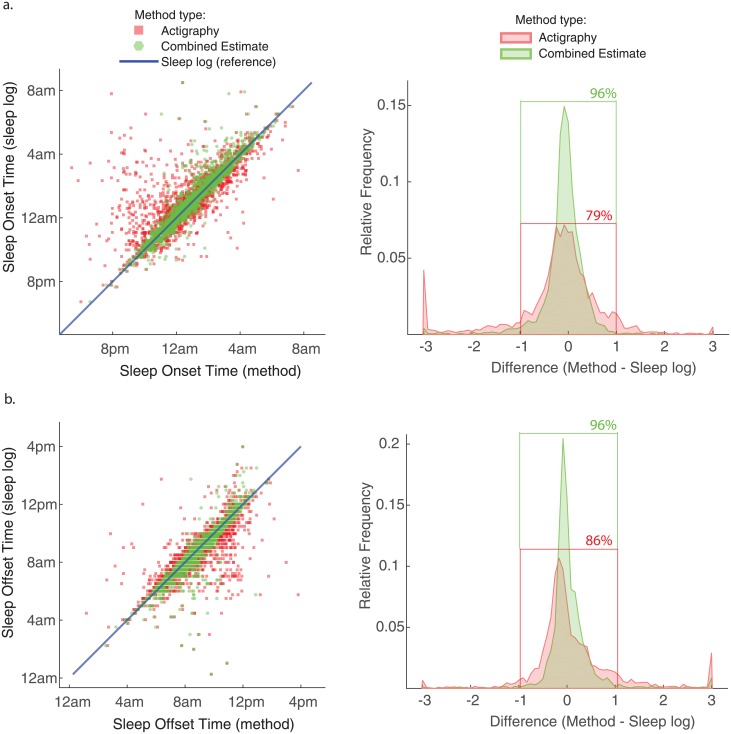
Narrowing of difference distribution and outlier reduction for combined estimates. Bivariate scatter plots for (a) sleep onset time, and (b) sleep offset time, showing actigraphy measurements (red) and combined estimates (green) with reference to sorted daily sleep log measurements (blue) across the entire data set. The difference distributions (right) are much sharper for combined estimates, with corresponding increase in agreement (e.g. a higher percentage of differences contained within ±1.0 hour).

We examined the relationship between actigraphy and combined estimates of SON and SOFF with reference to daily sleep log measurements (Fig 5). The scatter plots show an increase in agreement for the combined estimate (green markers) with reference to sleep log measurements (e.g. less dispersion from the blue reference line) by comparison to actigraphy measurements (red markers). We observe the largest influence of the model on SON (Fig 5a) for many of the early evening (prior to 10pm) actigraphy measurements, primarily by shifting them toward sleep log measurements that happened to be closer to the mean of sleep history. Likewise, a subset of actigraphy measurements for SOFF were substantially later than sleep logs (Fig 5b), and many of these values are found to be shifted toward the sleep log measurements. The combined estimate yielded a distinct sharpening of the difference distributions (Fig 5, green curves), in which 96% of differences were contained within the reference interval of ±1.0 hours compared to 79% and 86% of differences for SON and SOFF, respectively, derived from actigraphy alone. The output of this model had the desired effect of reducing large discrepancies between the measurements, which is practically useful for quantifying sleep history metrics to a singular value each day. Theoretically, the combined estimate should reduce measurement error associated with each modality and produce better estimates of the true sleep history of the individual.

## Discussion

Despite an extensive body of research studying acute episodes of sleep deprivation in the laboratory [14,52,53], much less is known about how chronic patterns of naturalistic sleep loss can impact variability in the functioning of an individual across various time scales including days, weeks, or even months. While epidemiological studies have found extensive links between sleep health and physical and mental health [54,55], these studies are not designed to isolate direct or causal links between sleep and behavior on a daily or weekly basis. Longitudinal sleep studies have the potential to advance our understanding of whether daily sleep measurements can help explain or predict intrinsic fluctuations in human cognition, behavior, or performance.

The current study measured variables associated with sleep and wake states derived from wrist actigraphy and daily sleep log questionnaires for up to four consecutive months from 30 individuals in their natural sleeping environment. It is important to recognize the limitations associated with inferential techniques for measuring naturalistic sleep and which aspects of sleep/wake behavior can and cannot be validly measured. Sleep stages are defined by specific changes in mental state, and as such, measuring the microstructure of sleep (i.e. precise transitions among sleep states) requires polysomnography, which measures neural and physiological signatures associated with specific sleep stages [21–23]. However, PSG is not currently a practical option for longitudinal studies of naturalistic sleep. Instead, these studies must rely upon inferential methods including questionnaires [25], wrist actigraphy [28–30] and/or other types of physiological signals [56–58] to estimate variables associated with sleep *macrostructure* (i.e. whether the individual was asleep or awake).

### Convergence in measuring sleep onset and offset times

Our results provide converging evidence for the ability of wrist actigraphy and sleep logs to accurately measure sleep onset and sleep offset times. In fact, a majority of differences between actigraphy and sleep log measurements were within a reasonable range of ±0.5 hours (56% for sleep onset and 65% for sleep offset). Yet, the difference distributions for all variables were also characterized by rather bulky tails, reflecting the fact that on some occasions the two methods also provided extremely divergent estimates. For example, absolute differences that were greater than two hours corresponded to 7.9% of the data for SON and 5.7% for SOFF.

The exact reason for such divergence on this subset of measurements is not readily determined due to the possibility of several contributing factors. There were many cases for SON in which actigraphy produced measurements that were substantially earlier in the evening than those provided by self-report. Across the entire data set, 8.1% of actigraphy measurements reported SON before 10:00pm, whereas only 2.5% of sleep logs reported SON before 10:00pm, and many of these measurements (36%) actually showed a substantial difference (> 3 hrs) with respect to self-report. Further, despite the strong correlation measured across all of the data, there was not a significant correlation between actigraphy and sleep logs for this particular subset of the data (*r*(62) = -0.13, *p* = 0.29), suggesting that these discrepancies were not systematic and were possibly due to issues with the actigraphy algorithm in incorrectly scoring evening rest periods (e.g., while reading a book or watching TV) as sleep. In these cases, it would be sensible to defer to measurements provided by self-report, especially when the self-reported times were more consistent with the overall sleep history of the individual.

Some of the other cases of large discrepancy could be due to individuals misreporting the time that they fell asleep or woke up, either due to a failure of memory or a lack of effort in completing the sleep log each day. Over the course of four months, it is plausible that for some individuals, the daily completion of a sleep log could become a tedious task, resulting in coarser self-estimates of sleep and wake behavior. This raises the possibility that sleep log compliance on an individual level could be an indicator for the reliability of data self-reported in sleep logs.

### Compliance as a trait-like factor for reliability of self-reported data

Strict compliance is important for studying sleep history because the lack of compliance in submitting sleep logs will result in missing data. Likewise, delaying the submission of sleep logs past the moment of awakening would likely introduce noise or variance to retrospective estimates of sleep/wake variables associated with the previous night [50]. Non-compliance is a particularly salient issue for sleep diaries [49] because completing a daily diary creates an extra burden on participants, who have to take time out of their day to fill out the questionnaire at the instructed time. In fact, we found that compliance in submitting sleep logs tended to worsen significantly over time according to a linear function, revealing a specific limitation associated with relying solely upon sleep logs for long-term sleep measurement. By contrast, strict compliance for wrist actigraphy is arguably much simpler because it only requires participants to wear the device correctly and make sure that the battery is charged.

There were, however, notable individual differences in compliance rates across subjects. Although we found that 60% of participants completed at least 75% of their required sleep log questionnaires, 13% of participants were found to complete less than 60%. We found a statistically significant relationship between compliance and agreement, showing that participants with higher overall compliance also tended to have higher levels of agreement. Due to the fact that sleep logs necessarily rely upon subjectivity and memory for events preceding the self-report by several hours (e.g. the time in bed the night before), it is reasonable to assume that differences in motivation or related factors, manifested through compliance behavior, could play a role in the precision and accuracy of sleep/wake estimations [59].

Consistent with this interpretation, we examined two personality trait questionnaires and discovered a significant and specific relationship between compliance and behavioral avoidance (BIS). The behavioral avoidance or inhibition system is posited to regulate motivation to aversive stimuli, including the goal to avoid unpleasant events and the production of negative affect [43]. Individuals with high behavioral inhibition tend to show more restraint and timid behaviors in response to new objects and situations, and have a greater tendency for mood and anxiety disorders [60]. The specific relationship between compliance and behavioral avoidance trait is an interesting finding that warrants further investigation., These results recommend caution in analyzing sleep history solely from sleep logs, especially those derived from individual participants demonstrating poor overall compliance to the study requirements.

### Lack of convergence for wakefulness

While we found strong convergence for SON and SOFF across the two methods, we also found a lack of convergence for sleep variables associated with wakefulness including SOL, WASO, and NNA. This result is consistent with much of the existing literature showing that wakefulness as measured by wrist actigraphy is typically of greater magnitude than subjective reports of wakefulness [39,61–64]. Yet, research directly comparing actigraphy to PSG has found that actigraphy may actually tend to underestimate the amount of true wakefulness [35,36], suggesting that sleep logs may even provide a dramatic underestimation of night wakefulness.

For longitudinal studies that seek to characterize the macrostructure of sleep using both actigraphy and sleep logs, the lack of convergence on wake-related variables precludes the ability to combine these independent estimates into a single robust estimate. Instead, empirical studies and quantitative models designed to examine statistical relationships between wake behavior at night and other outcome measures (performance, health, behavior, etc.), should consider SOL, WASO, and other sleep quality metrics separately for data acquired through wrist actigraphy and sleep logs. On the other hand, the strong agreement we observed for SON and SOFF theoretically permits development of statistical methods to combine these independent measurements into a single estimate.

### Combining actigraphy and sleep log measurements

Sleep onset and sleep offset are very important for characterizing the macrostructure of sleep. Precise measurements for the time of day that an individual fell asleep and woke up is useful for measuring circadian rhythms and the amount of time in bed, which also helps to quantify sleep opportunity and constrain estimates of total sleep time. We observed strong agreement (< 0.5 hours of absolute difference) for a majority of measurements for SON and SOFF, but there was also a subset of data (about 6–8% of total data) for which the two measurements differed substantially (> 2.0 hours of absolute difference), and it would not be reasonable to simply take the average for these discordant data points. To handle both cases of concordant and discordant measurement, we proposed a model that computes a weighted average of the two measurements with a built-in bias for measurements with higher probability given the empirically measured sleep history of the individual.

The output of this model produced combined estimates that had a much more narrow difference distribution, and reduced the number of highly discrepant measurements with reference to sleep log measurements (Fig 5). We expect that the combined estimates should provide a more accurate reflection of the true sleep variables experienced by individuals during the study. However, future work will be needed to statistically evaluate this hypothesis by comparing actigraphy and sleep log measurements to a gold standard such as PSG. Specifically, we predict that the combined estimate will show better agreement with PSG recorded sleep than actigraphy or sleep logs alone. Nonetheless, future research examining long-term naturalistic sleep history will benefit from a better understanding of when and where actigraphy and sleep logs tend to agree and disagree in measuring sleep/wake variables, how individual differences in compliance may play a role in overall data quality obtained by subjective reports, and how the two modalities may be combined in a principled way to potentially increase robustness and reduce noise, or measurement error, associated with the two distinct measurement tools.

## Supporting information

S1 File Excel file with the complete within-subjects data set. The spreadsheet includes for each day and for each subject the sleep-related variables measured by sleep logs and wrist actigraphy, as well as compliance data. Sheet 1 has definitions for variable headings in the table and relevant descriptions. For reference, between-subjects variables are reported in Table 2.
(XLSX)

## References

[1] Alhola P, Polo-Kantola P. Sleep Deprivation: Impact on Cognitive Performance. Neuropsychiatr Dis Treat. 2007;3: 553–567. 19300585 PMC2656292

[2] Killgore WDS. Effects of Sleep Deprivation on Cognition. Prog Brain Res. 2010;185: 105–129. doi: 10.1016/B978-0-444-53702-7.00007-5 21075236 10.1016/B978-0-444-53702-7.00007-5

[3] Killgore WDS, Grugle NL, Balkin TJ. Gambling When Sleep Deprived: Don’t Bet on Stimulants. Chronobiol Int. 2012;29: 43–54. doi: 10.3109/07420528.2011.635230 22217100 10.3109/07420528.2011.635230

[4] Walker MP. The Role of Sleep in Cognition and Emotion. Ann N Y Acad Sci. 2009;1156: 168–197. doi: 10.1111/j.1749-6632.2009.04416.x 19338508 10.1111/j.1749-6632.2009.04416.x

[5] Wong ML, Lau EYY, Wan JHY, Cheung SF, Hui CH, MOK DSY. The Interplay Between Sleep and Mood in Predicting Academic Functioning, Physical Health and Psychological Health: A Longitudinal Study. J Psychosom Res. 2013;74: 271–277. doi: 10.1016/j.jpsychores.2012.08.014 23497826 10.1016/j.jpsychores.2012.08.014

[6] Dinges DF, Pack F, Williams K, Gillen KA, Powell JW, Ott GE, et al Cumulative Sleepiness, Mood Disturbance, and Psychomotor Vigilance Performance Decrements During a Week of Sleep Restricted to 4–5 Hours Per Night. Sleep. 1997;20: 267–277. 9231952

[7] Doran SM, Van Dongen HPA, Dinges DF. Sustained Attention Performance During Sleep Deprivation: Evidence of State Instability. Arch Ital Biol. 2001;139: 253–267. 11330205

[8] Patanaik A, Kwoh CK, Chua ECP, Gooley JJ, Chee MWL. Classifying Vulnerability to Sleep Deprivation Using Baseline Measures of Psychomotor Vigilance. Sleep. 2015;38: 723–734. doi: 10.5665/sleep.4664 25325482 10.5665/sleep.4664 PMC4402656

[9] Roca J, Fuentes LJ, Marotta A, López-Ramón M-F, Castro C, Lupiáñez J, et al The Effects of Sleep Deprivation on the Attentional Functions and Vigilance. Acta Psychol (Amst). 2012;140: 164–176. doi: 10.1016/j.actpsy.2012.03.007 22634265 10.1016/j.actpsy.2012.03.007

[10] Chee MWL, Chuah LYM, Venkatraman V, Chan WY, Philip P, Dinges DF. Functional Imaging of Working Memory Following Normal Sleep and After 24 and 35 H of Sleep Deprivation: Correlations of Fronto-Parietal Activation with Performance. NeuroImage. 2006;31: 419–428. doi: 10.1016/j.neuroimage.2005.12.001 16427321 10.1016/j.neuroimage.2005.12.001

[11] Baum KT, Desai A, Field J, Miller LE, Rausch J, Beebe DW. Sleep Restriction Worsens Mood and Emotion Regulation in Adolescents. J Child Psychol Psychiatry. 2014;55: 180–190. doi: 10.1111/jcpp.12125 24889207 10.1111/jcpp.12125 PMC4047523

[12] Beattie L, Kyle SD, Espie CA, Biello SM. Social interactions, emotion and sleep: A systematic review and research agenda. Sleep Med Rev. 2015;24: 83–100. doi: 10.1016/j.smrv.2014.12.005 25697832 10.1016/j.smrv.2014.12.005

[13] Cho K, Barnes CM, Guanara CL. Sleepy Punishers Are Harsh Punishers: Daylight Saving Time and Legal Sentences. Psychol Sci. 2016; 0956797616678437. doi: 10.1177/0956797616678437 28182529

[14] Durmer JS, Dinges DF. Neurocognitive Consequences of Sleep Deprivation. Semin Neurol. 2005;25: 117–129. doi: 10.1055/s-2005-867080 15798944 10.1055/s-2005-867080

[15] Ferrara M, De Gennaro L. How much sleep do we need? Sleep Med Rev. 2001;5: 155–179. doi: 10.1053/smrv.2000.0138 12531052 10.1053/smrv.2000.0138

[16] Haack M, Mullington JM. Sustained sleep restriction reduces emotional and physical well-being. Pain. 2005;119: 56–64. doi: 10.1016/j.pain.2005.09.011 16297554 10.1016/j.pain.2005.09.011

[17] Luyster FS, Strollo PJ, Zee PC, Walsh JK. Sleep: A Health Imperative. Sleep. 2012;35: 727–734. doi: 10.5665/sleep.1846 22654183 10.5665/sleep.1846 PMC3353049

[18] Spiegel K, Leproult R, Van Cauter E. Impact of sleep debt on metabolic and endocrine function. Lancet Lond Engl. 1999;354: 1435–1439. doi: 10.1016/S0140-6736(99)01376-8 10.1016/S0140-6736(99)01376-8 10543671

[19] Moturu ST, Khayal I, Aharony N, Pan W, Pentland A. Using Social Sensing to Understand the Links between Sleep, Mood, and Sociability. 2011 IEEE Third International Conference on Privacy, Security, Risk and Trust and 2011 IEEE Third International Conference on Social Computing. 2011. pp. 208–214.

[20] Totterdell P, Reynolds S, Parkinson B, Briner RB. Associations of sleep with everyday mood, minor symptoms and social interaction experience. Sleep J Sleep Res Sleep Med. 1994;17: 466–475. 10.1093/sleep/17.5.466 7991960

[21] Loomis AL, Harvey EN, Hobart GA. Cerebral states during sleep, as studied by human brain potentials. J Exp Psychol. 1937;21: 127–144.

[22] Monk TH, Buysse DJ, Rose LR. Wrist Actigraphic Measures of Sleep in Space. Sleep. 1999;22: 948–954. doi: 10.1093/sleep/22.7.948 10566913

[23] Penzel T, Conradt R. Computer based sleep recording and analysis. Sleep Med Rev. 2000;4: 131–148. doi: 10.1053/smrv.1999.0087 12531163 10.1053/smrv.1999.0087

[24] Carney CE, Lajos LE, Waters WF. Wrist Actigraph Versus Self-Report in Normal Sleepers: Sleep Schedule Adherence and Self-Report Validity. Behav Sleep Med. 2004;2: 134–143. doi: 10.1207/s15402010bsm0203_2 15600229 10.1207/s15402010bsm0203_2

[25] Monk TH, Reynolds CF, Kupfer DJ, Buysse DJ, Coble PA, Hayes AJ, et al The Pittsburgh Sleep Diary. J Sleep Res. 1994;3: 111–120. doi: 10.1111/j.1365-2869.1994.tb00114.x 10607115

[26] Jean-Louis G, Kripke DF, Cole RJ, Assmus JD, Langer RD. Sleep detection with an accelerometer actigraph: comparisons with polysomnography. Physiol Behav. 2001;72: 21–28. 11239977 10.1016/s0031-9384(00)00355-3

[27] Martin JL, Hakim AD. Wrist Actigraphy. CHEST J. 2011;139: 1514–1527. doi: 10.1378/chest.10-1872 21652563 10.1378/chest.10-1872 PMC3109647

[28] Ancoli-Israel S, Cole R, Alessi C, Chambers M, Moorcroft W, Pollak CP. The role of actigraphy in the study of sleep and circadian rhythms. Sleep J Sleep Sleep Disord Res. 2003; Available: http://psycnet.apa.org/psycinfo/2003-05243-015 10.1093/sleep/26.3.342 12749557

[29] Sadeh A. The role and validity of actigraphy in sleep medicine: An update. Sleep Med Rev. 2011;15: 259–267. doi: 10.1016/j.smrv.2010.10.001 21237680 10.1016/j.smrv.2010.10.001

[30] Tryon WW. Issues of validity in actigraphic sleep assessment. Sleep. 2004;27: 158–165. 14998254 10.1093/sleep/27.1.158

[31] Cellini N, Buman MP, McDevitt EA, Ricker AA, Mednick SC. Direct comparison of two actigraphy devices with polysomnographically recorded naps in healthy young adults. Chronobiol Int. 2013;30: 691–698. doi: 10.3109/07420528.2013.782312 23721120 10.3109/07420528.2013.782312

[32] Kaplan KA, Talbot LS, Gruber J, Harvey AG. Evaluating sleep in bipolar disorder: comparison between actigraphy, polysomnography, and sleep diary. Bipolar Disord. 2012;14: 870–879. doi: 10.1111/bdi.12021 23167935 10.1111/bdi.12021 PMC3549461

[33] Lauderdale DS, Knutson KL, Yan LL, Liu K, Rathouz PJ. Sleep Duration: How Well Do Self-Reports Reflect Objective Measures? The CARDIA Sleep Study. Epidemiol Camb Mass. 2008;19: 838–845. 10.1097/EDE.0b013e318187a7b0 PMC2785092 18854708

[34] Meltzer LJ, Walsh CM, Traylor J, Westin AML. Direct Comparison of Two New Actigraphs and Polysomnography in Children and Adolescents. Sleep. 2012;35: 159–166. doi: 10.5665/sleep.1608 22215930 10.5665/sleep.1608 PMC3242684

[35] Pollak CP, Tryon WW, Nagaraja H, Dzwonczyk R. How accurately does wrist actigraphy identify the states of sleep and wakefulness? Sleep. 2001;24: 957–965. 11766166 10.1093/sleep/24.8.957

[36] de Souza L, Benedito-Silva AA, Pires MLN, Poyares D, Tufik S, Calil HM. Further validation of actigraphy for sleep studies. Sleep. 2003;26: 81–85. 12627737 10.1093/sleep/26.1.81

[37] Van de Water ATM, Holmes A, Hurley DA. Objective measurements of sleep for non-laboratory settings as alternatives to polysomnography—a systematic review. J Sleep Res. 2011;20: 183–200. doi: 10.1111/j.1365-2869.2009.00814.x 20374444 10.1111/j.1365-2869.2009.00814.x

[38] Hedner J, Pillar G, Pittman SD, Zou D, Grote L, White DP. A Novel Adaptive Wrist Actigraphy Algorithm for Sleep-Wake Assessment in Sleep Apnea Patients. Sleep. 2004;27: 1560–1566. doi: 10.1093/sleep/27.8.1560 15683148 10.1093/sleep/27.8.1560

[39] Lockley SW, Skene DJ, Arendt J. Comparison between subjective and actigraphic measurement of sleep and sleep rhythms. J Sleep Res. 1999;8: 175–183. doi: 10.1046/j.1365-2869.1999.00155.x 10476003 10.1046/j.1365-2869.1999.00155.x

[40] Auger RR, Varghese R, Silber MH, Slocumb NL. Total sleep time obtained from actigraphy versus sleep logs in an academic sleep center and impact on further sleep testing. Nat Sci Sleep. 2013;5: 125–131. doi: 10.2147/NSS.S48970 24124399 10.2147/NSS.S48970 PMC3794964

[41] Sadeh A. Assessment of intervention for infant night waking: parental reports and activity-based home monitoring. J Consult Clin Psychol. 1994;62: 63–68. 8034831 10.1037//0022-006x.62.1.63

[42] Goldberg LR. The structure of phenotypic personality traits. Am Psychol. 1993;48: 26–34. 8427480 10.1037//0003-066x.48.1.26

[43] Carver CS, White TL. Behavioral Inhibition, Behavioral Activation, and Affective Responses to Impending Reward and Punishment: The Bis/bas Scales. J Pers Soc Psychol. 1994;67: 319–333.

[44] Russell C, Caldwell JA, Arand D, Myers L, Wubbels P, Downs H. Validation of the Fatigue Science Readiband Actigraph and Associated Sleep/Wake Classification Algorithms. Arch LLC. 2000;

[45] Driller M, McQuillan J, O’Donnell S. Inter-device reliability of an automatic-scoring actigraph for measuring sleep in healthy adults. Sleep Sci. 2016;9: 198–201. doi: 10.1016/j.slsci.2016.08.003 28123660 10.1016/j.slsci.2016.08.003 PMC5241607

[46] Caia J, Thornton HR, Kelly VG, Scott TJ, Halson SL, Cupples B, et al Does self-perceived sleep reflect sleep estimated via activity monitors in professional rugby league athletes? J Sports Sci. 2017;0: 1–5. doi: 10.1080/02640414.2017.1398885 29087784 10.1080/02640414.2017.1398885

[47] Adler AB, Gunia BC, Bliese PD, Kim PY, LoPresti ML. Using actigraphy feedback to improve sleep in soldiers: an exploratory trial. Sleep Health. 2017;3: 126–131. doi: 10.1016/j.sleh.2017.01.001 28346159 10.1016/j.sleh.2017.01.001

[48] Berger AM, Wielgus KK, Young-McCaughan S, Fischer P, Farr L, Lee KA. Methodological Challenges When Using Actigraphy in Research. J Pain Symptom Manage. 2008;36: 191–199. doi: 10.1016/j.jpainsymman.2007.10.008 18400460 10.1016/j.jpainsymman.2007.10.008 PMC2542506

[49] Stone AA, Shiffman S, Schwartz JE, Broderick JE, Hufford MR. Patient compliance with paper and electronic diaries. Control Clin Trials. 2003;24: 182–199. doi: 10.1016/S0197-2456(02)00320-3 12689739 10.1016/s0197-2456(02)00320-3

[50] Stone AA, Shiffman S. Capturing momentary, self-report data: A proposal for reporting guidelines. Ann Behav Med. 2002;24: 236–243. doi: 10.1207/S15324796ABM2403_09 12173681 10.1207/S15324796ABM2403_09

[51] Bland JM, Altman DG. Measuring agreement in method comparison studies. Stat Methods Med Res. 1999;8: 135–160. doi: 10.1177/096228029900800204 10501650 10.1177/096228029900800204

[52] Banks S, Dinges DF. Behavioral and Physiological Consequences of Sleep Restriction. J Clin Sleep Med JCSM Off Publ Am Acad Sleep Med. 2007;3: 519–528. PMC1978335 17803017

[53] Boonstra TW, Stins JF, Daffertshofer A, Beek PJ. Effects of sleep deprivation on neural functioning: an integrative review. Cell Mol Life Sci. 2007;64: 934 doi: 10.1007/s00018-007-6457-8 17347797 10.1007/s00018-007-6457-8 PMC2778638

[54] Sivertsen B, Krokstad S, Øverland S, Mykletun A. The epidemiology of insomnia: Associations with physical and mental health.: The HUNT-2 study. J Psychosom Res. 2009;67: 109–116. doi: 10.1016/j.jpsychores.2009.05.001 19616137 10.1016/j.jpsychores.2009.05.001

[55] Young T, Peppard PE, Gottlieb DJ. Epidemiology of obstructive sleep apnea: a population health perspective. Am J Respir Crit Care Med. 2002;165: 1217–1239. 11991871 10.1164/rccm.2109080

[56] Chen Z, Lin M, Chen F, Lane ND, Cardone G, Wang R, et al. Unobtrusive sleep monitoring using smartphones. 2013 7th International Conference on Pervasive Computing Technologies for Healthcare and Workshops. 2013. pp. 145–152.

[57] Kortelainen JM, van Gils M, Pärkkä J. Multichannel bed pressure sensor for sleep monitoring. 2012 Computing in Cardiology. 2012 pp. 313–316.

[58] Nishyama M, Miyamoto M, Watanabe K. Respiration and body movement analysis during sleep in bed using hetero-core fiber optic pressure sensors without constraint to human activity. J Biomed Opt. 2011;16: 017002 doi: 10.1117/1.3528008 21280923 10.1117/1.3528008

[59] Green AS, Rafaeli E, Bolger N, Shrout PE, Reis HT. Paper or plastic? Data equivalence in paper and electronic diaries. Psychol Methods. 2006;11: 87–105. doi: 10.1037/1082-989X.11.1.87 16594769 10.1037/1082-989X.11.1.87

[60] Muris P, Brakel van AML, Arntz A, Schouten E. Behavioral Inhibition as a Risk Factor for the Development of Childhood Anxiety Disorders: A Longitudinal Study. J Child Fam Stud. 2011;20: 157–170. doi: 10.1007/s10826-010-9365-8 21475710 10.1007/s10826-010-9365-8 PMC3048305

[61] Iwasaki M, Iwata S, Iemura A, Yamashita N, Tomino Y, Anme T, et al Utility of Subjective Sleep Assessment Tools for Healthy Preschool Children: A Comparative Study Between Sleep Logs, Questionnaires, and Actigraphy. J Epidemiol. 2010;20: 143–149. doi: 10.2188/jea.JE20090054 20139658 10.2188/jea.JE20090054 PMC3900813

[62] Matsumoto K, Shinkoda H, Kang MJ, Seo YJ. Longitudinal Study of Mothers’ Sleep-Wake Behaviors and Circadian Time Patterns from Late Pregnancy to Postpartum–Monitoring of Wrist Actigraphy and Sleep Logs. Biol Rhythm Res. 2003;34: 265–278. doi: 10.1076/brhm.34.3.265.18812

[63] Short MA, Gradisar M, Lack LC, Wright H, Carskadon MA. The discrepancy between actigraphic and sleep diary measures of sleep in adolescents. Sleep Med. 2012;13: 378–384. doi: 10.1016/j.sleep.2011.11.005 22437142 10.1016/j.sleep.2011.11.005

[64] So K, Michael Adamson T, Horne RSC. The use of actigraphy for assessment of the development of sleep/wake patterns in infants during the first 12 months of life. J Sleep Res. 2007;16: 181–187. doi: 10.1111/j.1365-2869.2007.00582.x 17542948 10.1111/j.1365-2869.2007.00582.x

